# Therapeutic efficacy of SYM004, a mixture of two anti-EGFR antibodies in human colorectal cancer with acquired resistance to cetuximab and MET activation

**DOI:** 10.18632/oncotarget.18749

**Published:** 2017-06-27

**Authors:** Stefania Napolitano, Giulia Martini, Erika Martinelli, Valentina Belli, Alessia Parascandolo, Mikko O. Laukkanen, Vincenzo Sforza, Floriana Morgillo, Davide Ciardiello, Fortunato Ciardiello, Teresa Troiani

**Affiliations:** ^1^ Oncologia Medica, Dipartimento Medico-Chirurgico di Internistica Clinica e Sperimentale “F. Magrassi e A. Lanzara”, Università degli Studi della Campania Luigi Vanvitelli, 80131 Naples, Italy; ^2^ IRCCS SDN, Via Gianturco, 80143 Naples, Italy

**Keywords:** SYM004, cetuximab, acquired resistance, MET, metastatic colorectal cancer

## Abstract

**Purpose:**

Cetuximab and panitumumab have an effective therapeutic response in a subset of *RAS* Wild-Type (WT) metastatic colorectal cancers (mCRCs). Despite molecular-driven selection, all patients do not respond to epidermal growth factor receptor (EGFR) inhibitors and the onset of secondary resistance limits their clinical benefit.

**Experimental Design:**

We tested, *in vitro* and *in vivo*, the effect of SYM004, a 1:1 mixture of two recombinant human-mouse chimeric monoclonal antibodies (mAbs) directed against non-overlapping epitopes of the EGFR, on CRC models with acquired resistance to cetuximab.

**Results:**

SYM004 showed a potent growth inhibitory effect in CRC cell lines with acquired resistance to cetuximab and MET activation. SYM004 treatment determined a significant induction of apoptosis and a strong inhibition of MET, AKT and MAPK phosphorilation in these resistant models. The data may further suggest SYM004 -driven induced internalization and degradation of the antibody-receptor complex, which prevents cross-interaction between EGFR and MET even in the presence of TGFα. Moreover, *in vivo* xenograft studies demonstrated that SYM004 has stronger antitumor activity than cetuximab in CRC models. Importantly, in the current work we observed a response to therapy in all cetuximab resistant tumors mice treated with SYM004. More importantly, four out of seven mice continue to respond to SYM004 after 30 weeks of treatment underling the prolonged effect of the drug.

**Conclusion:**

These results suggest that the treatment with SYM004 could be a strategy to overcome acquired resistance to first generation of anti-EGFR therapies in mCRC as a result of MET activation.

## Translational relevance

EGFR is an attractive target for anti-cancer therapy. Despite the clinical success of cetuximab and panitumumab, the efficacy of these agents is limited by development of acquired resistance. Several therapeutic strategies designed to circumvent resistance driven by downstream pathway reactivation are being investigated in ongoing clinical trials combining anti-EGFR drug with other targeted therapies. However, it remains a significant unmet need for a therapeutic strategy to overcome acquired resistance to anti-EGFR mAbs. SYM004 has already shown to be effective in some cetuximab-resistant CRC models. However, other mechanisms of secondary resistance also need to be investigated to define the patient population potentially benefiting from SYM004. In the present study, we have evaluated the *in vitro* and *in vivo* activity of SYM004 in human CRC models with acquired resistance to cetuximab.

## INTRODUCTION

Colorectal cancer (CRC) is the third most commonly diagnosed cancer and the fourth most common cause of cancer-related death [1]. Using improvements in knowledge of colon cancer biology, new drugs targeting specific pathways important for carcinogenesis, metastasis, proliferation and angiogenesis have been incorporated in metastatic CRC (mCRC) treatment strategies. In this scenario, epidermal growth factor receptor (EGFR) is an attractive target for anticancer therapy. The epidermal growth factor receptors are a family of trans-membrane receptor tyrosine kinases, which includes EGFR or HER1, HER2, HER3 and HER4. These receptors play an important role in normal cell growth, metabolism, proliferation, survival, and differentiation. However, deregulation through mutation, overexpression, or gene amplification of the HER family is commonly associated with development, progression, or acquired resistance to therapies in several human cancers [2]. Homo- or hetero-dimerization induced by binding of ligands within the EGF family of growth factors results in cross-phosphorylation of the dimerization partners, ultimately triggering intracellular signaling, including the RAS-RAF-MEK-ERK and the PI3K-AKT axes [3, 4]. Such downstream signaling pathways are primarily involved in cell proliferation, differentiation, apoptosis and cell invasion [2–4].

Cetuximab and panitumumab are two monoclonal antibodies (mAbs) that, by targeting the extracellular domain of the EGFR, inhibit ligand binding, receptor dimerization and subsequent activation of downstream intracellular signaling pathways [3]. Based on the results of randomized clinical trials, these two mAbs have been approved for treatment of *RAS* Wild-Type (WT) mCRC patients [5, 6].

Clinical treatment of mCRC is challenged by development of acquired drug resistance. Patients, who initially show therapeutic response to e.g. EGFR mAbs, may have a relapse of the disease caused by additional mutations with consequent development of drug resistance. [7, 8]. The field of acquired resistance, thought preclinical and clinical data, has gained a central role in the last few years, with the emergence of new insights. Various mechanisms have been described as responsible for acquired resistance: the most common event is the emergence of *KRAS*, *NRAS* and *BRAF* mutations [7–9]. Such mutations presumably are either present in a clonal subpopulation within the tumor before treatment initiation or rise as a consequence of continued mutagenesis over the course of therapy. In the absence of alterations in *RAS* or its immediate downstream effectors, other mechanisms have been involved in the activation of the EGFR pathway. Genetic aberrations in tyrosine kinase receptors (TKRs), such as HER2 and MET, have been shown to bypass EGFR signaling, activate the MAPK cascade and, therefore to confer acquired resistance to anti-EGFR therapies [10–13]. Moreover, after EGFR blockade, about 20% of CRC patients develop mutations in the EGFR extracellular domain (ECD) that impair antibody binding and are associated with clinical relapse [14, 15]. The observed alterations in oncogene and signal transduction activities demonstrate molecular complexity of the late phase metastatic cancers suggesting various alternative survival mechanisms for cancer cells and reflecting the high level of molecular heterogeneity.

Several strategies have been developed in order to circumvent resistance to anti-EGFR mAbs. In particular, preclinical studies have demonstrated that combination of targeted treatments that leads to a vertical inhibition of the EGFR pathway is one of the possible approaches [16–18].

SYM004 is a 1:1 mixture of two recombinant human-mouse chimeric mAbs directed against non-overlapping epitopes of the EGFR [19]. The binding site of the two antibodies is different from cetuximab, and, therefore, SYM004 could be effective even in presence of mutations in the ECD of the EGFR [20]. Characteristically SYM004 induces EGFR internalization into the cytoplasmic compartment with consequent inactivation of EGFR by cross-linking. As previously shown, the combination of two antibodies targeting non overlapping epitopes on EGFR act synergistic and superior to individual antibodies in terms of target elimination and cancer cell growth inhibition [21]. The inactivation of EGFR by SYM004 causes significantly inhibited receptor activity, markedly reduced EGFR cell surface expression, and significantly reduced EGFR heterodimer formation as compared to individual antibodies, such as cetuximab [22, 23].

The aim of this study was gain insights on the efficacy of SYM004 to circumvent cetuximab resistance in several CRC models with acquired resistance to cetuximab that we have previously characterized in our laboratory [12, 16–18].

## RESULTS

### Effects of cetuximab and SYM004 on cell proliferation and induction of apoptosis in a panel of human colorectal cancer cell lines

**Figure 1:**
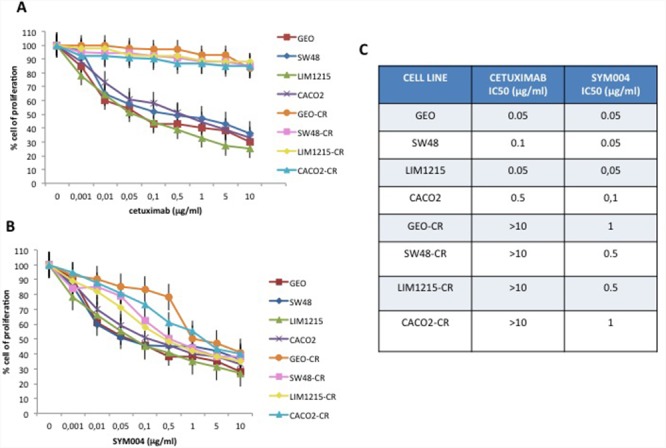
Effects of cetuximab or SYM004 treatment on cell proliferation in a panel of human CRC cell lines **(A-B)** Cells were treated with different concentrations of cetuximab (range, 0.001 to 10 μg/ml) and SYM004 (range, 0.001 to 10 μg/ml) for 96 hours and evaluated for proliferation by MTT staining, as described in Materials and Methods. **(C)** The IC_50_ was determined by interpolation from the dose-response curves. Results represent the median of three separate experiments, each performed in quadruplicate.

In the last few years we have developed models that could help to better understand the molecular mechanisms of acquired resistance to anti-EGFR inhibitors. In particular, we have generated human CRC cells with acquired resistance to cetuximab, such as GEO-CR and SW48-CR as previously described [12, 16–18]. To further expand this panel of human colon cancer cell lines, we have selected other two CRC cells, sensitive to EGFR blockade, such as CACO2 and LIM1215, to generate by an *in vitro* selection new models of acquired resistance to cetuximab (CACO2-CR and LIM1215-CR). These two cell lines have been chosen because of their mutational profile with no mutations in *KRAS, NRAS, BRAF* and *PIK3CA* genes. After the establishment of EGFR resistant cell lines we have characterized their resistant phenotype by cell proliferation analysis using a MTT assay in the presence of cetuximab. Cancer cells were treated with cetuximab at concentrations ranging from 0.001 to 10 μg/ml and with SYM004 at concentrations ranging from 0.001 to 10 μg/ml for 96 hours. The drug concentrations required to inhibit cell growth by 50% (IC_50_) were determined by interpolation from the dose-response curves. As illustrated in Figure 1A and 1C, we observed 10-fold increase in the IC_50_ in cetuximab-resistant cancer cell lines as compared with parental cells. To evaluate the potency and efficacy of SYM004 in inhibiting CRC cell growth, we have performed a cell proliferation analysis. As depicted in Figure 1B and 1C, no significant difference in efficacy between SYM004 and cetuximab was observed among cetuximab-sensitive CRC cells. On the contrary, SYM004 shows a potent anti-proliferative effect in cetuximab-resistant CRC cells with IC_50_ values approximately 10 times less than the IC_50_ of cetuximab (Figure 1A-1C).

**Figure 2:**
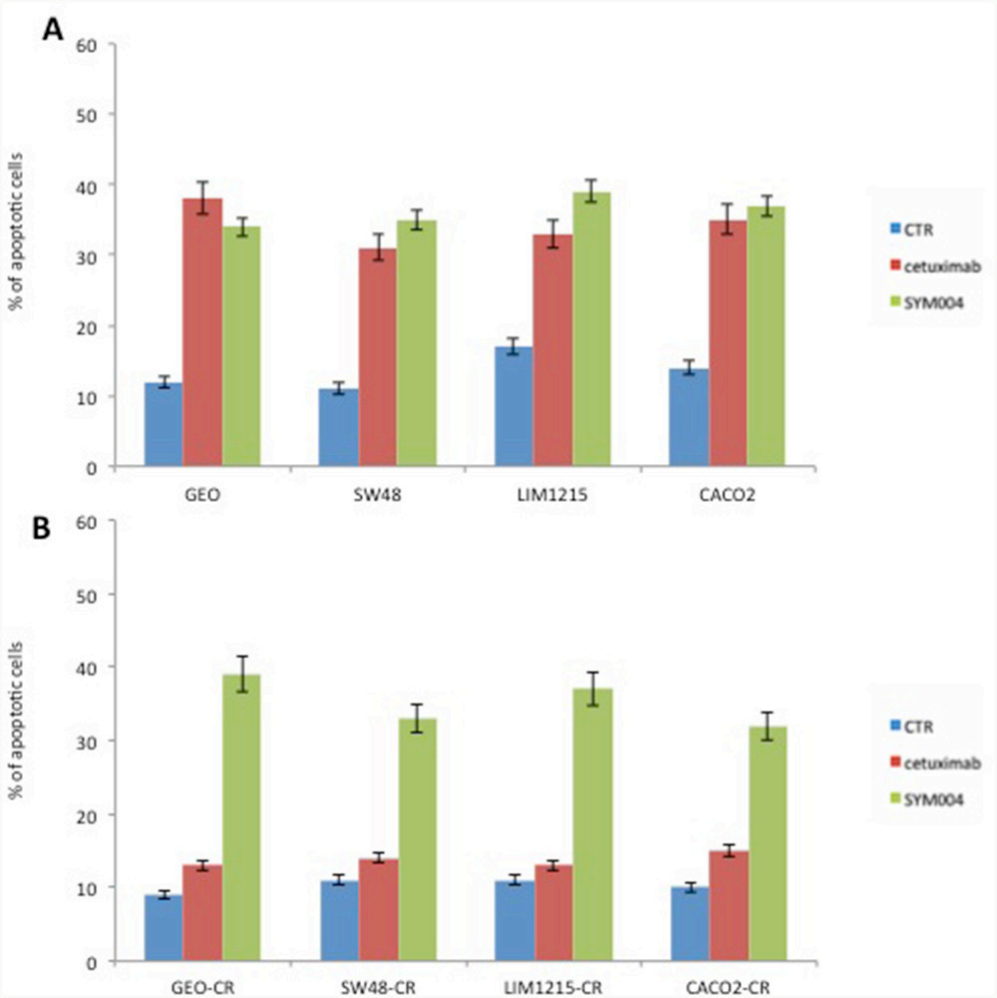
Effects of cetuximab or SYM004 in induction of apoptosis in a panel of human colorectal cancer cell lines **(A-B)** Cetuximab-sensitive and resistant CRC cells were treated for 48 hours with the 5 μg/ml of cetuximab or SYM004. Apoptosis was evaluated with Annexin V staining, as described in Materials and Methods and the rate of apoptosis was expressed as a percentage of the total cells counted.

Next, we measured the ability of cetuximab and SYM004 to induce apoptosis by using Annexin V-FITC. As depicted in Figure 2A-2B, both drugs determined a significant induction of apoptosis in cetuximab-sensitive CRC cell lines, whereas in cetuximab-resistant cells only SYM004 treatment resulted in significantly increased apoptotic cell death.

### Effects of cetuximab and SYM004 on EGFR-dependent intracellular signalling in a panel of human colorectal cancer cell lines

**Figure 3:**
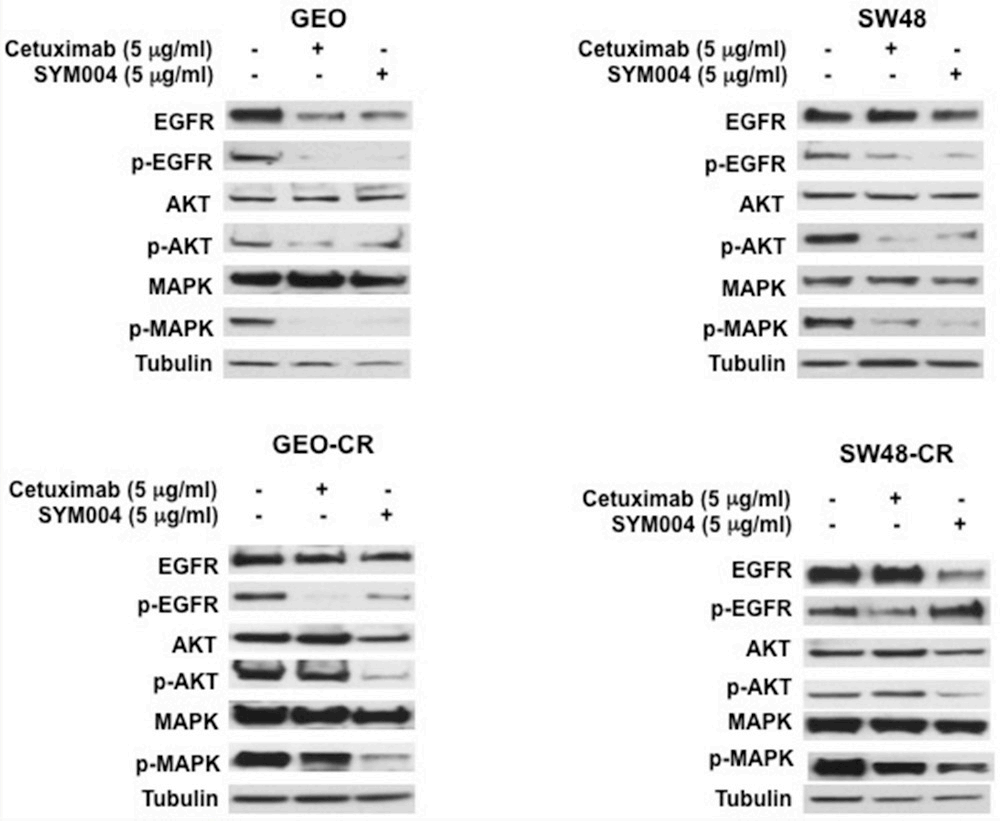
Effects of cetuximab or SYM004 on EGFR-dependent intracellular signaling in a panel of human colorectal cancer cell lines Cells were treated with cetuximab and SYM004 at the indicated doses for 24 hrs. Total cell protein extracts (50μg) were subjected to immunoblotting with the indicated antibodies, as described in Materials and Methods. Anti-tubulin antibody was used for normalization of protein extract content. Experiments were repeated three times.

Further, to determinate the effect of these mAbs on EGFR and its downstream signaling pathway, we have selected two cetuximab-resistant cancer cells (GEO-CR and SW48-CR) and their parental cell lines. Western blot analysis revealed that the level of EGFR decreased in all four SYM004 treated cells, whereas no decrease in EGFR levels was seen in cells treated with cetuximab. The cetuximab treatment resulted in inhibition of phosphorylated MAPK and AKT proteins only in the cetuximab-sensitive cell lines, whereas no reduction was observed in the cetuximab-resistant cells (Figure 3). On the contrary, the anti-proliferative activity of SYM004 was coupled by inhibition of MAPK and AKT phosphorylation in all four cancer cell lines (Figure 3). All these findings suggested that SYM004 could overcome resistance to anti-EGFR treatment by inhibiting PIK3CA/AKT and MAPK pathways in CRC cancer cells with acquired resistance to cetuximab.

### Effect of SYM004 in human colorectal cancer models with acquired resistance to cetuximab such as MET activation and ERBB2 amplification

**Figure 4:**
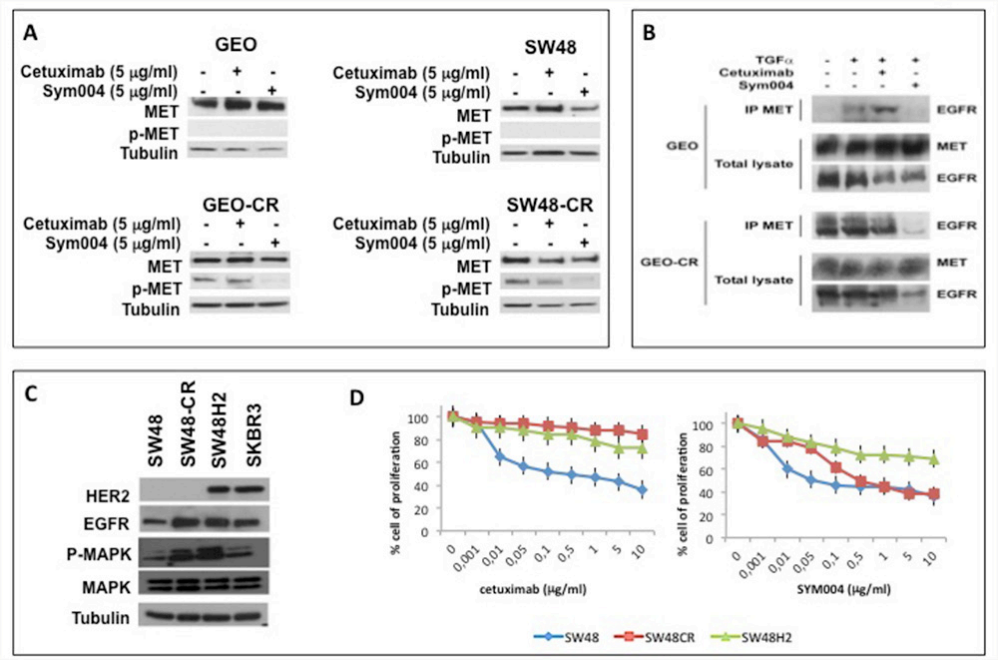
Effects of cetuximab or SYM004 in human colorectal cancer cell lines with acquired resistance to cetuximab such as MET activation and ERBB2 amplification **(A)** Western blot analysis of protein expression in GEO, SW48, GEO-CR and SW48-CR cells treated with cetuximab (5 μg/ml) and SYM004 (5 μg/ml) was performed. Total cell protein extracts were subjected to immuneblotting with the indicated antibodies, as described in Materials and Methods. **(B)** Two mg of GEO cell or of GEO-CR cell protein extracts were immune-precipitated with a specific anti-MET antibody and then were immune-blotted with a specific anti-EGFR antibody, as described in Materials and Methods. **(C)** Western blot analysis of protein expression in SW48, SW48-CR, SW48H2 and SKBR3 was performed. Total cell protein extracts were subjected to immuneblotting with the indicated antibodies, as described in Materials and Methods. **(D)** HER2 gene amplified SW48 cells line (SW48H2) are exposed to different concentration of cetuximab (range, 0.001 to 10 μg/ml) and SYM004 (range, 0.001 to 10 μg/ml) for 96 hours and evaluated for proliferation by MTT staining, as described in Materials and Methods.

We have previously described that in cetuximab-resistant cancer cells (GEO-CR, SW48-CR) cell proliferation and survival pathways are activated by MET [12, 13]. In order to evaluate the effect of SYM004 on MET phosphorylation we performed Western blot analysis. As depicted in Figure 4A, SYM004 induces a significant reduction on MET phosphorylation in both cetuximab-resistant CRC cell lines. We have previously demonstrated that enhanced expression of the selective EGFR ligand transforming growth factor α (TGFα) in cetuximab-resistant CRC cells is responsible for EGFR-MET interaction and subsequent MET phosphorylation and activation [12]. We hypothesize that the anti-proliferative effects of SYM004 on cetuximab-resistant cells, are related to the ability of the mAbs to cross-link the EGFR by causing internalization and subsequent degradation of the antibody-receptor complex. In fact, the reduction of EGFR expression on cell surface does not allow the hetero-dimerization with MET and subsequently its activation. To demonstrate this hypothesis we performed a co-immunoprecipation analysis (Figure 4B). As shown in Figure 4B, EGFR immunoprecipitated together with MET in GEO-CR cells, but not in GEO cells. Moreover, to elucidate the potential role of TGFα in inducing EGFR-MET interaction, GEO, GEO-CR and SW48, SW48-CR (data not shown) cells were treated with TGFα in the presence or in the absence of cetuximab and SYM004. Lysates were immunoprecipitated with anti-MET antibody and then assayed by western blotting with anti-EGFR antibody. As reported in Figure 4B, TGFα treatment induced EGFR-MET heterodimerization in GEO cells and this effect is potentiated in GEO-CR cells. The EGFR-MET heterodimerization was also observed following combined treatment with TGFα and cetuximab in both sensitive and resistant cells but not in presence of SYM004, suggesting that, the effect of SYM004 to induce internalization and subsequent degradation of the antibody-receptor complex could be responsible of the failure cross-interaction between EGFR and MET even in presence of TGFα.

The activation and/or amplification of MET are one of the possible acquired resistance mechanisms to anti-EGFR treatment in mCRC. Another mechanism of anti-EGFR resistance is *HER2* amplification [10, 11]. To determinate the capability of SYM004 to overcome intrinsic resistance to cetuximab related to *HER2* amplification, we have transfected SW48 cells with *HER2* genes. Transfection of the *HER2* gene result in cells stably overexpressing the HER2 protein (Figure 4C). We have exposed HER2 gene amplified SW48 cells line (SW48H2) to different concentration of cetuximab and SYM004. We have characterized their resistant phenotype by cell proliferation analysis using a MTT assay. As shown in Figure 4D, SYM004 has similar antiproliferative effects to cetuximab in these cells lines. These results suggest that SYM004 could not be able to overcome resistance to cetuximab related to HER2 overexpression.

### Effects of cetuximab and SYM004 on human colorectal cancer tumor xenograft models

**Figure 5:**
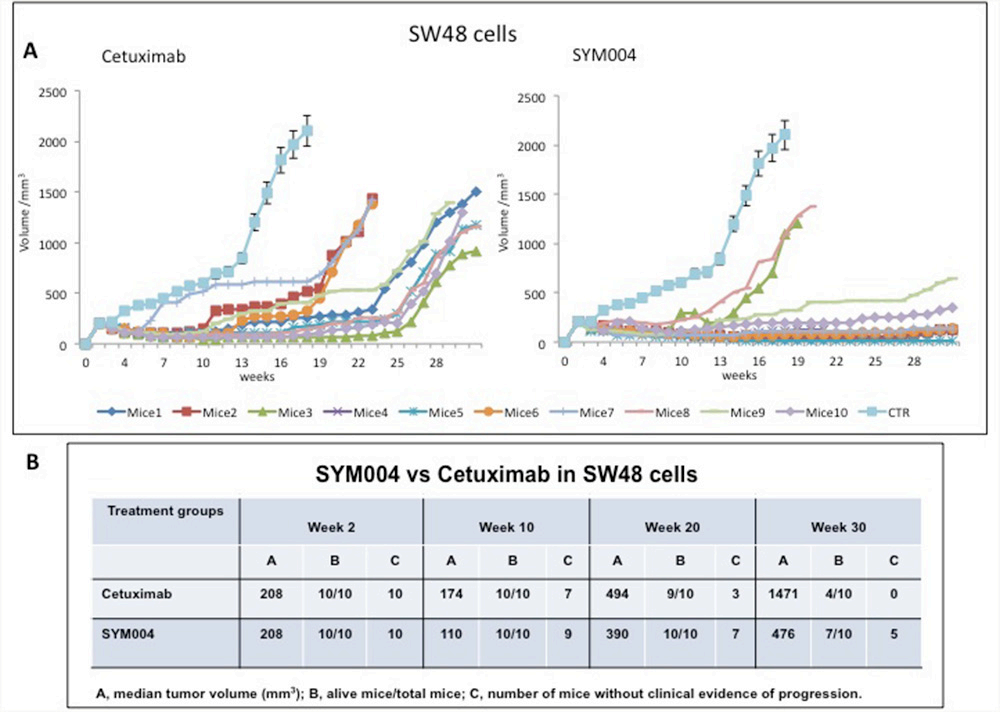
Effects of cetuximab or SYM004 on SW48 tumor xenografts **(A-B)** Mice were injected subcutaneously in the right flank with SW48 cells as described in the Materials and Methods. After two weeks (average tumor size 200-300 mm^3^) mice were treated intraperitoneally with: PBS control, cetuximab (1 mg twice a week), SYM004 (50 mg/kg twice a week). The treatment was continued for 30 weeks. Each group consisted of 10 mice. Tumor volumes were measured three times a week. Animals were sacrificed when tumors achieved 2.000 mm^3^ in size. Abbreviations: CTR, control; A, median tumor volume (mm^3^); B, alive mice/total mice; C, number of mice without clinical evidence of progression.

To investigate the antitumor activity of SYM004 *in vivo* we injected SW48, LIM1215 and CACO2 cell lines subcutaneously to female nude mice. Mice were randomly assigned to receive vehicle, cetuximab or SYM004 for 30 weeks. As shown in Figure 5A-5B initially both cetuximab and SYM004 demonstrated similar suppression of tumor growth until week 10, after which treatment with SYM004 provided stronger growth inhibition compared to cetuximab demonstrating almost complete suppression of tumor growth (Figure 5A-5B, Supplementary Figure 1A-1B and Supplementary Figure 2A-2B). Importantly, after 10 weeks of treatment the median tumor volume in the SYM004 treated group was only 110 mm^3^ and one out of 10 mice demonstrated progression of disease, whereas in cetuximab treated group tumor volume was 174 mm3 and three out of ten mice showed increased tumor growth (Figure 5A-5B). A similar difference was observed in LIM1215 and CACO2 xenograft models (Supplementary Figure 1A-1B and Supplementary Figure 2A-2B). Moreover, the antitumor activity of SYM004 was prolonged until 30 weeks of treatment in all three xenograft models. At week 30, the median tumor volume in the SW48 xenograft, was 476 mm^3^ in the SYM004 treatment group with five out of ten mice without progression of disease and seven out of ten mice still alive. On the contrary, in the cetuximab treatment group at week 30 median tumor volume was 1471 mm^3^ with all mice experiencing disease progression and with only four out of ten mice being still alive (Figure 5A-5B). A similar differential antitumor efficacy was observed in LIM1215 and CACO2 xenograft models. (Supplementary Figure 1A-1B and Supplementary Figure 2A-2B). Furthermore, no recurring tumors were detected in any of SYM004-treated mice with complete response, demonstrating a prolonged treatment response.

**Figure 6:**
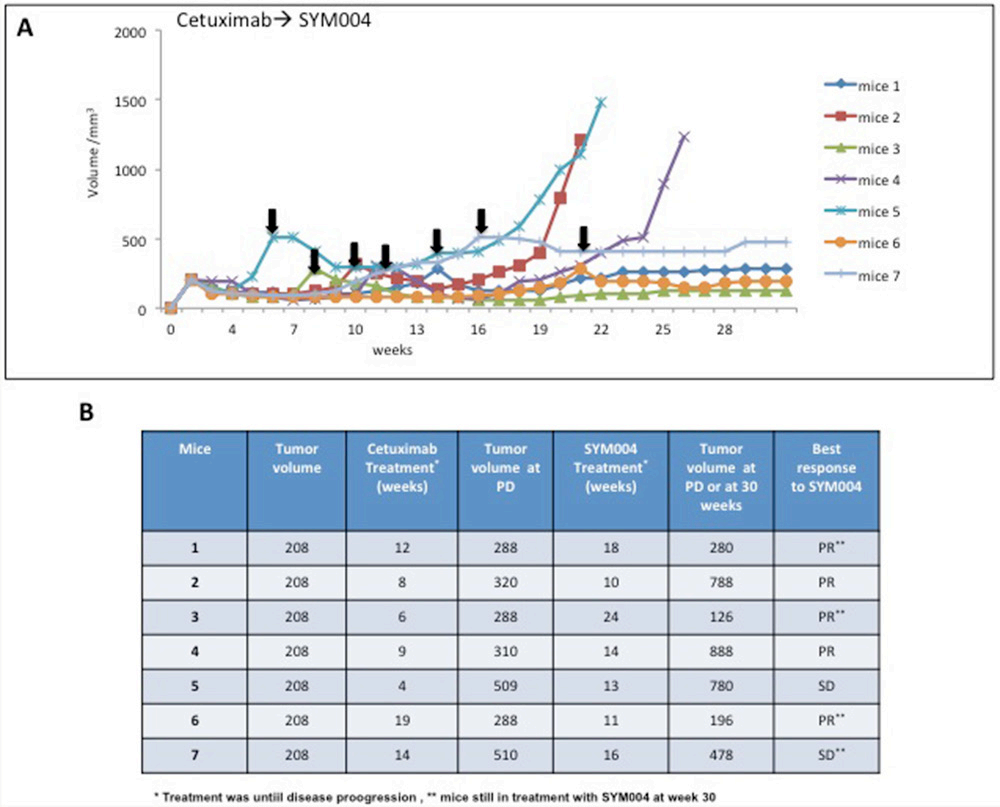
Effect of SYM004 treatment after progression to Cetuximab therapy in SW48 tumor xenografts **(A-B)** SW48 cells were injected s.c. into the right flank of seven nude mice. After two weeks mice were treated with Cetuximab (1 mg twice a week) by i.p. injection. Treatment was continued until disease progression. The black arrows indicate the time of progression to cetuximab. At progression phase mice were assigned to SYM004 treatment (50 mg/Kg twice a week) by i.p. injection. The treatment was continued until 30 weeks. At week 30 four out of seven mice were still responding to SYM004 (as indicated by double asterisk). Abbreviations: PD, progression disease; PR, partial response; SD, stable disease.

To further dissect the effect of SYM004 on cetuximab resistant cells *in vivo* we transplanted SW48 cells in nude mice and let the tumor to grow 100 mm^3^ before initiating drug treatments. Mice were initially treated with two weekly i.p. doses of cetuximab until grafted cells demonstrated increased growth. At disease progression phase the drug was changed to SYM004. To monitor tumor response to therapy, we measured volumetric changes and used an arbitrary classification method partially based on clinical practice as described in Material and Methods. As shown in Figure 6A-6B, in all seven mice treatment with SYM004 at progression phase of the disease provided further antitumor activity. All mice received treatment with SYM004 for at least 10 weeks. Importantly, four out of seven mice at week 30 continue to respond to SYM004 treatment, underlying the prolonged effect of the drug. The treatment with SYM004 induced five partial responses and two stable diseases, achieving 100% disease control rate. The delayed tumor growth in the SYM004 group was accompanied by a prolonged survival that was significantly different compared with cetuximab group (data not shown).

Both treatment protocols were well tolerated by mice and were not accompanied by any major side effect or treatment-related weight loss. No cellular abnormalities were observed in the examined organs, including heart, lung, liver, kidney and spleen derived from all xenograft mouse models (data not shown).

## DISCUSSION

The commonly observed activation of EGFR and downstream signal transduction pathways in cancers is caused by increased ligand expression, genomic amplification of EGFR, and heterodimer formation of EGFR with other RTKs [24–27]. Changes in EGFR status have been linked to the development and maintenance of a malignant phenotype and correlated to poor clinical prognosis [28]. For this reason, EGFR is an attractive target for anti-cancer therapy [2, 3]. Cetuximab and panitumumab are two mAbs approved for the treatment of *RAS* WT mCRC [5, 6]. Cetuximab is a monoclonal chimeric (mouse–human) IgG1 antibody targeting the domain III of the extracellular part of the EGFR, whereas panitumumab is a fully human IgG2 antibody binding to slightly different epitopes of the extracellular domain of the EGFR [29].

Despite the clinical success of cetuximab and panitumumab, the efficacy of these agents is limited by development of acquired resistance [7, 8], which is caused by activation of canonical and non-canonical signal transduction pathways [10–13, 30]. It is noteworthy that emerging new mutations are observed in molecules mediating EGFR signaling (EGFR, KRAS, NRAS, and BRAF) indicating the key role of EGFR as an upstream signal transduction drug target [7, 9].

Although several targeted therapeutic strategies designed to circumvent resistance driven by downstream pathway reactivation are being investigated in ongoing clinical trials that combine anti-EGFR drug with other targeted therapies [16, 17, 31, 32], additional therapeutic strategies are required to overcome resistance to anti-EGFR therapies and improve overall survival in m CRC patients. Based on the recent data, combination therapy preclinical trials utilizing antibodies targeted to non-overlapping epitopes might offer substantially more efficient inhibition of aberrant RTK activation and downregulation of tumor growth. [19, 33, 34]. Therefore, we hypothesized that SYM004, a novel anti-EGFR mAbs mixture, may overcome resistance to cetuximab. SYM004 consists of two anti-EGFR antibodies that bind two non-overlapping epitopes on domain III of the EGFR inducing efficient internalization and inactivation of EGFR with consequent inhibition of cellular proliferation [19]. There is emerging *in vitro* and *in vivo* experimental evidence suggesting superiority of SYM004 to first-generation anti-EGFR antibodies, such as cetuximab and panitumumab, in a wide range of cancers [19–21, 35]. Furthermore, SYM004 has shown activity in some cancer cells with acquired resistance to cetuximab [22]. Sanchez-Martin FJ et al. have evaluated the efficacy of SYM004, *in vitro* and *in vivo*, in cetuximab-resistant CRC models and have found that SYM004 is a valid strategy to treat CRC tumors harboring EGFR ECD mutations [20]. In addition, Dienstmann et al. have shown that SYM004 is highly efficient in blocking CRC cell growth in the presence of the high-affinity ligands EGF and TGFα, factors known to be both upregulated in response to anti-EGFR antibody treatment and potential determinants of EGFR inhibitor resistance [23]. All these data suggest a superior antitumor effect of SYM004 in comparison with cetuximab that was specifically evident in cells showing EGFR ligand–dependent growth.

The activity of SYM004 is also under investigation in randomized clinical trial. In the Phase II clinical trial, the efficacy of SYM004 was valuated in *RAS* WT mCRC patients previously treated with anti-EGFR therapy progressing within 6 months from trial enrolment (NCT02083653 ClinicalTrials.gov Identifier). The trial compared SYM004 versus investigator's choice (Fluorouracil 5 FU or capecitabine monotherapy). The study design is based on preliminary results of two previous Phases I trial. In the first one (SYM004-01), after enrollment of 11 patients, the most frequent toxicities observed were skin rash, hypomagnesemia and hypocalcemia (NCT01117428 ClinicalTrials.gov Identifier). In the second Phase I trial, SYM004 achieved disease control in 26 of 39 enrolled patients with acquired resistance to anti-EGFR therapy. Skin toxicity and hypomagnesemia events of grade 3 or higher were reported for 50% and 21% of the patients, respectively, whereas only 3% of the patients experienced diarrhea of grade 3 or higher [23].

However, other mechanisms of secondary resistance also need to be investigated to define the patient population potentially benefiting from SYM004. In a previous study, we have demonstrated that resistance to cetuximab in CRC cells could be mediated by TGFα overexpression that induced EGFR-MET interaction with subsequent MET pathway activation. Blockade of both EGFR and MET receptor tyrosine kinases could represent a strategy for preventing and/or overcoming cetuximab resistance in these CRC models [12]. We further hypothesized that the pharmacodynamics effects of SYM004, with sustained decrease in EGFR expression and with the effective blockade of ligand–receptor interaction together with receptor down modulation, may explain the antiproliferative activity of SYM004 in cetuximab-resistant CRC models. In this respect, here we suggest that the reduction of EGFR on cancer cell membrane, could not allow EGFR hetero-dimerization with MET and, therefore, subsequently block MET transactivation.

Another mechanism responsible of anti-EGFR resistance is *HER2* amplification [10, 11]. To determinate the capability of SYM004 to overcome resistance induced by HER2 amplification, we have transfected SW48 cells with *HER2*. However, SYM004 was not effective in this resistant CRC model. Yonesaka et al. have shown, in patients with acquired resistance to cetuximab, that *HER2* amplification present in a small percentage of pretreatment tumor cells (14 %) increase considerably in post treatment samples (71 %) [10]. These results indicate that enhanced HER2 signaling could confer both primary and acquired resistance. The reason of the lack of activity of SYM004 could be explained on the basis of different acquired resistance mechanisms induced by HER2 amplification that are not dependent on direct EGFR signaling pathway perturbations. Thus, SYM004 could not be an effective treatment option for all mCRC with resistance to anti-EGFR antibodies, such as cetuximab or panitumumab.

In the current work the activity of SYM004 was further explored in *in vivo* CRC models of acquired resistance to cetuximab. Nude mice were subcutaneously injected with SW48, LIM1215 or CACO2 cell lines and were randomly assigned to receive vehicle, cetuximab or SYM004 for 30 weeks. SYM004 demonstrated a stronger antitumor activity and prolonged effect in these CRC models resulting in significantly smaller tumor volumes as compared to cetuximab treated mice. After 10 weeks of treatment, evidence of clinical progression was evident in only one (for SW48 and LIM1215 xenografts) or two (for CACO2 xenografts) out of ten mice in SYM004 treatment group. On the contrary, in the cetuximab treatment group, the evidence of the clinical progression was evident in five out of ten mice (for LIM1215 and CACO2 xenografts) and in three out of ten mice (for SW48 xenografts). At week 30 the prolonged effect of SYM004 was more pronounced. Interestingly, in SYM004 treatment group five SW48 xenograft mices, six LIM1215 xenograft mices and seven CACO2 xenograft mices were still responding to the treatment. In contrast, in the cetuximab treated group all mice showed progression of disease except for the CACO2 xenografts, in which one out of ten mice was still responding to the treatment.

Furthermore, in all mice whose tumors were resistant to cetuximab and that were subsequently treated with SYM004, responses to therapy were observed. In fact, four out of seven mice, at week 30 continue to respond to SYM004 treatment, underlying the prolonged effect of the drug. This result indicates that SYM004 may be effective in tumors that are resistant to cetuximab.

Collectively, the results of the present study support the evidence for the EGFR pathway as an important target for therapeutic intervention in WT *RAS* mCRC beyond treatment with first generation mAbs, such as cetuximab. A subgroup of mCRC could remain “EGFR-addicted” despite progression on anti-EGFR treatment. SYM004, a mixture of two mAbs that do not overlap for EGFR binding with cetuximab or panitumumab, is a potential effective therapeutic option in this setting.

## MATERIALS AND METHODS

### Drugs

Cetuximab, an anti-EGFR human-mouse chimeric monoclonal antibody was kindly provided by Merck Serono Italy (Rome, Italy). SYM004 was kindly provided by Symphogen A/S (Lyngby, Denmark). For *in vitro* and *in vivo* applications, cetuximab and SYM004 were ready to use and they were stored refrigerated (2^0^ C-8^0^ C) in the dark until use.

### Cell lines

The human SW48 (catalogue number: HTL99020) (*KRAS, NRAS, BRAF* and *PIK3CA* WT), colon cancer cell line was obtained from IRCCS “Azienda Ospedaliera Universitaria San Martino-IST Istituto Nazionale per la Ricerca sul Cancro, Genova” Italy. The human GEO [*KRAS* mutation (G12A); *NRAS, BRAF*, and *PIK3CA* WT] colon cancer cell line was kindly provided by Dr. N. Normanno (National Cancer Institute, Naples, Italy). The human LIM 1215 (*KRAS, NRAS, BRAF* and *PIK3CA* WT), colon cancer cell line was obtained from Dr.ssa Di Nicolantonio at Candiolo National Cancer Institute (Candiolo, Italy). The human CACO2 (*KRAS, NRAS, BRAF* and *PIK3CA* WT), colon cancer cell line was obtained from Dr. A. Fiorentino at Department of Environmental Biological and Pharmaceutical Sciences and Technologies, Second University of Naples, (Caserta, Italy). GEO-CR and SW48-CR cells were established, as previously described [12, 16–18]. To generate cetuximab-resistant LIM1215 and CACO2 cancer cell lines, over a period of 6 months these cells were continuously exposed to increasing concentrations of cetuximab. The starting dose was the dose causing the inhibition of 50% of cancer cell growth (IC_50_). The drug dose was progressively increased to 1μg/ml in approximately 2 months, to 5 μg/ml after other 2 months and, finally, to 10 μg/ml after additional 2 months. The established cetuximab-resistant LIM1215 and CACO2 cancer cell lines (LIM1215-CR and CACO2 CR) were then maintained in continuous culture with this maximally achieved dose of cetuximab that allowed cellular proliferation. GEO, GEO-CR, CACO2 and CACO2-CR cell lines were grown in McCoy culture medium (Lonza, Cologne, Germany), supplemented with 10% fetal bovine serum (FBS) (Lonza), 1% penicillin/streptomycin (Lonza). SW48, SW48-CR, LIM 1215 and LIM1215-CR cells were grown in RPMI-1640 culture medium (Lonza) supplemented with 10% FBS, 1% penicillin/streptomycin. All cell lines were grown in a humidified incubator with 5% of carbon dioxide (CO_2_) and 95% air at 37°C. All cell lines were routinely screened for the presence of mycoplasma (Mycoplasma Detection Kit, Roche Diagnostics, Monza, Italy).

### Proliferation assay

Cancer cell lines were seeded in 24-well plates and were treated with different concentrations of cetuximab (range, 0.001 to 10 μg/ml) and SYM004 (range, 0.001 to 10 μg/ml) for 96 hours. Cell proliferation was measured with the 3-(4,5-dimethylthiazol-2-yl)-2,5-diphenyltetrazolium bromide (MTT). The IC_50_ was determined by interpolation from the dose-response curves. Results represent the median of three separate experiments, each performed in quadruplicate.

### Apoptosis assay

GEO, SW48, LIM1215, CACO2 cells and the cetuximab-resistant cell lines GEO-CR, SW48-CR, LIM1215-CR and CACO2-CR were seeded in six-well plates, treated with cetuximab and SYM004 at different concentrations as indicated for 72 hours and stained with Annexin V-fluorescein isothiocynate (FITC) (Invitrogen, CA, USA). Apoptotic cell death was assessed by counting the numbers of cells that stained positive for Annexin V-FITC using an Apoptosis Annexin V-FITC Kit (Invitrogen, CA, USA), coupled with fluorescence-activated cell sorting (FACS) analysis, by following manufacturer's protocol.

### Western blotting and immunoprecipitation

GE0, SW48, GEO-CR and SW48-CR cells were seeded into 100 mm^3^ dishes and treated with vehicle, cetuximab, SYM004 for 24 hours at different concentration as indicated. 50 μg of protein lysates, estimated by a modified Bradford assay (Bio-Rad, Munich, Germany), were subjected to Immunoprecipitation or Western blot by using the following antibodies: HERB2 monoclonal antibody (#2165), EGFR monoclonal antibody (#4267), phospho-EGFR monoclonal antibody (#3777), MET monoclonal antibody (#3127), phospho-MET monoclonal antibody (#3077), AKT policlonal antibody (#9272), phospho-AKT monoclonal antibody (#4060), p44/42 MAPK polyclonal antibody (#9102), phospho-p44/42MAPK monoclonal antibody (#9106) were from Cell Signaling (Beverly, MA, USA). Monoclonal anti-α-tubulin antibody (T8203) was from Sigma Chemical Co. (St. Louis, MO, USA). Secondary antibodies goat anti-rabbit IgG and rabbit anti-mouse IgG were from Bio-rad (Hercules, CA, USA). Immunoreactive proteins were visualized by enhanced chemiluminescence. (ECL plus, Thermo Fisher Scientific, Rockford, IL, USA). Each experiment was done in triplicate. For immunoprecipitation 2 mg of protein lysates were immune-precipitated with the required antibodies; immune-complexes were recovered with protein G Sepharose (Roche Diagnostics) and detected by Western blotting.

### Transfection of SW48 cells with *HER-2*

SW48 cells were transfected with 5μg of pcDNA3 HER2 plasmid (#16257, Addgene, Cambridge, MA, USA) using FuGENE® HD Transfection Reagent (Promega, Madison, WI, USA) following manufacturer's instructions. The day before transfection, cells were plated in 10-mm dishes at 40% of confluence in DMEM supplemented with 10% FBS. After 48 hours Geneticin® (GIBCO by Life Technologies, Thermo Fischer Scientific, Waltham, MA, USA) at final concentration of 1.25 mg/ml was used to select Geneticin–resistant mass population.

### Tumor xenografts in nude mice

Four- to six-week old female balb/c athymic (nu+/nu+) mice were purchased from Charles River Laboratories (Milan, Italy). The research protocol was approved and mice were maintained in accordance with the institutional guidelines of the Second University of Naples Animal Care and Use Committee. Animal care was in compliance with Italian (Decree 116/92) and European Community (E.C. L358/1 18/12/86) guidelines on the use and protection of laboratory animals. Mice were acclimatized at the Second University of Naples Medical School Animal Facility for 1 week prior to being injected with cancer cells and then caged in groups of five under controlled conditions (12–12 h light-dark cycle; room temperature 20±22°C; humidity 55–60%). A total number of 3.5 × 10^6^ SW48, LIM1215 and CACO-2 cells in 200 μl of matrigel (BD Biosciences, Milan, IT): PBS (1:1) were subcutaneously injected to the right flank of mice. When the mean values of tumors were between 200-300 mm^3^, mice were randomly assigned to one of the following groups (ten mice per group). Group 1: vehicles administrated intraperitoneally (i.p.). Group 2: cetuximab injected twice a week i.p. at the dose of 1 mg. Group 3: SYM004 administered twice a week i.p. at the dose of 50 mg/Kg. Monitoring of tumor growth was performed until tumors reached approximately 2.000 mm^3^, when mice were euthanized. The treatment was continued for 30 weeks. The mice body weights were monitored daily. Tumor size was evaluated twice a week by calliper measurements using the following formula: π/6 x larger diameter x (smaller diameter)^2^.

For assessment of tumor response to treatment, we used volume measurements and adopted a classification methodology loosely inspired by clinical criteria: (i) tumor regression (or shrinkage) was defined as a decrease of at least 50% in the volume of target lesions, taking as reference the baseline tumor volume; (ii) at least a 35% increase in tumor volume identified disease progression; and (iii) responses that were neither sufficient reduction to qualify for shrinkage or sufficient increase to qualify for progression were considered as disease stabilization.

### Statistical analysis

The statistical analyses of *in vitro* and *in vivo* data were carried out using Prism version 4.02 (GraphPad Software, Inc.). The Student t test was used to evaluate the statistical significance of the results. All P values represent 2-sided tests of statistical significance with P value < 0.05.

## SUPPLEMENTARY FIGURES


