# Supplementary material for: Therapeutic efficacy of SYM004, a mixture of two anti-EGFR antibodies in human colorectal cancer with acquired resistance to cetuximab and MET activation
Source: Oncotarget. 2017 Jun 27;8(40):67592–604. doi: 10.18632/oncotarget.18749 (PMC5620195; doi:10.18632/oncotarget.18749)
Supplement: Supplementary file 1 [file oncotarget-08-67592-s001.pdf]

Therapeutic efficacy of SYM004, a mixture of two anti-EGFR antibodies in human colorectal cancer with acquired resistance to cetuximab and MET activation

SUPPLEMENTARY MATERIALS

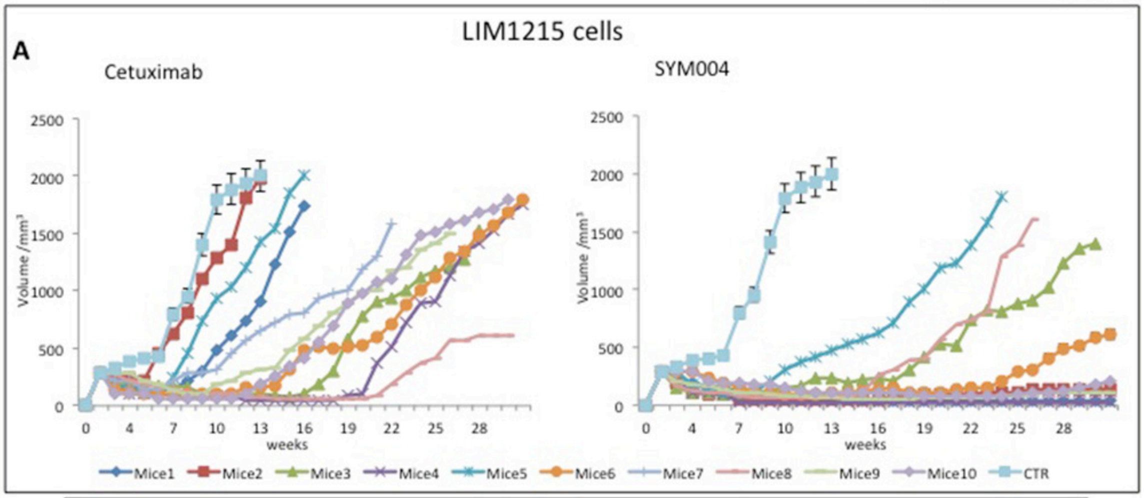

B

SYM004 vs Cetuximab in LIM1215 cells

Treatment groups	Week 2			Week 10			Week 20			Week 30		
	A	B	C	A	B	C	A	B	C	A	B	C
Cetuximab	290	10/10	10	416	10/10	5	1100	9/10	1	1628	3/10	0
SYM004	290	10/10	10	116	10/10	9	292	10/10	7	600	7/10	6

A, median tumor volume (mm^3); B, alive mice/total mice; C, number of mice without clinical evidence of progression.

Supplementary Figure 1: Effects of cetuximab and SYM004 on LIM1215 tumor xenografts. (A-B) Mice were injected subcutaneously in the right flank with SW48 cell lines as described in the Materials and Methods. After two weeks (average tumor size 200-300 mm^3) mice were treated intraperitoneally with: PBS control, cetuximab (1 mg twice a week), SYM004 (50 mg/kg twice a week). The treatment was continued for 30 weeks. Each group consisted of 10 mice. Tumor volumes were measured three times a week. Animals were sacrificed when tumors achieved 2.000 mm^3 in size. Abbreviations: CTR, control; A, median tumor volume (mm^3); B, alive mice/total mice; C, number of mice without clinical evidence of progression.

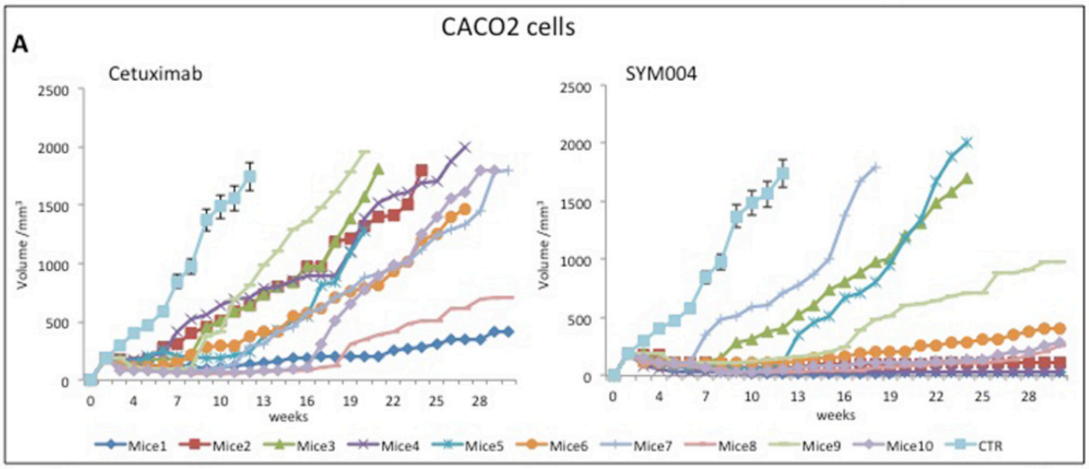

B

SYM004 vs Cetuximab in CACO2 cells

Treatment groups	Week 2			Week 10			Week 20			Week 30		
	A	B	C	A	B	C	A	B	C	A	B	C
Cetuximab	186	10/10	10	344	10/10	5	1114	8/10	1	1530	3/10	1
SYM004	186	10/10	10	151	10/10	8	566	9/10	6	766	7/10	7

A, median tumor volume (mm^3); B, alive mice/total mice; C, number of mice without clinical evidence of progression.

Supplementary Figure 2: Effects of cetuximab and SYM004 on CACO2 tumor xenografts. **(A-B)** Mice were injected subcutaneously in the right flank with SW48 cell lines as described in the Materials and Methods. After two weeks (average tumor size 200-300 mm^3) mice were treated intraperitoneally with: PBS (phosphate-buffered saline) control, cetuximab (1 mg twice a week), SYM004 (50 mg/kg twice a week). The treatment was continued for 30 weeks. Each group consisted of 10 mice. Tumor volumes were measured three times a week. Animals were sacrificed when tumors achieved 2.000 mm^3 in size. Abbreviations: CTR, control; A, median tumor volume (mm^3); B, alive mice/total mice; C, number of mice without clinical evidence of progression.
